# Transcriptome analysis reveals the regulation of cyclic nucleotide-gated ion channels in response to exogenous abscisic acid and calcium treatment under drought stress in tomato

**DOI:** 10.3389/fgene.2023.1139087

**Published:** 2023-02-28

**Authors:** Jinyan Shi, Xiangge Du

**Affiliations:** College of Plant Protection, China Agricultural University, Beijing, China

**Keywords:** cyclic nucleotide-gated ion channels family, drought, exogenous abscisic acid, exogenous Ca^2+^, RNA-sequencing, tomato

## Abstract

**Background:** Drought stress can limit the growth and development of tomato seedlings and cause considerable loss of tomato yield. Exogenous abscisic acid (ABA) and calcium (Ca^2+^) can effectively alleviate the damage of drought stress to plants in part because Ca^2+^ acts as a second messenger in the drought resistance pathway. Although cyclic nucleotide-gated ion channels (CNGCs) are common non-specific Ca^2+^ osmotic channels in cell membranes, a thorough understanding of the transcriptome characteristics of tomato treated with exogenous ABA and Ca^2+^ under drought stress is necessary to characterize the molecular mechanism of CNGC involved in tomato drought resistance.

**Results:** There were 12,896 differentially expressed genes in tomato under drought stress, as well as 11,406 and 12,502 differentially expressed genes after exogenous ABA and Ca^2+^ application, respectively. According to functional annotations and reports, the 19 SlCNGC genes related to Ca^2+^ transport were initially screened, with 11 SlCNGC genes that were upregulated under drought stress and downregulated after exogenous ABA application. After exogenous Ca^2+^ application, the data showed that two of these genes were upregulated, while nine genes were downregulated. Based on these expression patterns, we predicted the role of SlCNGC genes in the drought resistance pathway and their regulation by exogenous ABA and Ca^2+^ in tomato.

**Conclusion:** The results of this study provide foundational data for further study of the function of SlCNGC genes and a more comprehensive understanding of drought resistance mechanisms in tomato.

## 1 Introduction

Tomato (*Solanum lycopersicum*) is an important vegetable crop that is grown worldwide. China is the world’s largest tomato producer, accounting for nearly one-third of global output (Li et al., 2021). The tomato genome includes about 57,119 genes and is a rich resource for scientific study (Takei et al., 2021). Ca^2+^ is a ubiquitous second messenger in plant cells. It is a crucial medium for coupling extracellular signals with intracellular physiological responses and is closely related to the orderly operation of all life activities in plants (Berridge et al., 2000; Dodd et al., 2010). The regulation of cytosolic free Ca^2+^ concentration is an essential part of the signaling network of plant cells in response to environmental stimuli (McAinsh and Pittman, 2009; Edel et al., 2017). However, the channels encoded by the cyclic nucleotide-gated ion channels (CNGCs) gene family are ubiquitous, non-specific Ca^2+^ osmotic cation transporters present in the plant cell membrane (Zagotta and Siegelbaum, 1996; Zelman et al., 2012). Plant and animal CNGCs are highly similar in structure; the N- and C-termini of plant CNGCs are found on the inner side of the plasma membrane. The C-terminus contains a highly conserved cyclic nucleotide-binding domain (CNBD) and a calmodulin-binding domain (CaMBD) (Zelman et al., 2012). In plants, CNGCs proteins are mainly distributed in the cytoplasmic membrane and have later been found in mitochondrial, vacuolar, chloroplast thylakoid, and nuclear membranes (Gobert et al., 2006; Baxter et al., 2008).

Previous studies have reported that *Arabidopsis thaliana* CNGC genes have regulatory effects on a variety of plant biological pathways, and the molecular mechanism of this regulation has been identified. The protein, AtCNGC18 is involved in regulating pollen tube growth; its deletion leads to the failure of pollen tube formation in *Arabidopsis thaliana* and a lethal male sterility phenotype (Frietsch et al., 2007; Gao et al., 2014; Gao et al., 2016; Gu et al., 2017). The protein AtCNGC3 regulates the non-selective uptake of monovalent cations in *A. thaliana* root tissues and the *Atcngc3* mutant is more resistant to NaCl and KCl toxicity than wild-type seedlings (Gobert et al., 2006). The AtCNGC6 protein promotes the expression of the heat shock protein gene and the acquisition of heat tolerance by mediating heat-induced Ca^2+^ influx (Gao et al., 2012; Raja et al., 2019; Niu et al., 2020; Wang D et al., 2021). In addition to *A. thaliana*, CNGC genes also play important functions in other plant species. OsCNGC13 expression increases seed production by promoting pollen tube growth in style tissue (Moeder and Yoshioka, 2017; Xu et al., 2017). Furthermore, OsCNGC9 enhances the cold tolerance of rice by regulating the influx of Ca^2+^ into the cytoplasm in response to low temperatures (Wang J et al., 2021). The CNGC1, CNGC8, CNGC10, and CNGC20 genes in loquat may play a regulatory role in the drought resistance pathway (Wang W et al., 2021), and ZjCNGC2 may act as a negative regulator of the cold stress response in Chinese jujube by mediating signal transduction involving ZjMAPKK4 (Wang et al., 2020).

When plants are exposed to adverse stress, abscisic acid (ABA) accumulates to alleviate the damage caused by abiotic stresses such as high salt, drought, low temperature, and heat, which enhance the environmental adaptability of plants and improves their tolerance (Chinnusamy et al., 2008; Cutler et al., 2010; Fujita et al., 2011). The CBF/DREB1 transcription factors are expressed by low temperature and ABA induction in grape vegetative tissues and seedlings, which affect the expression of antioxidant and dehydrin genes, resulting in an increase in the cold tolerance of buds (Rubio et al., 2019). These two abiotic stresses have synergistic effects in the activation of these transcription factors (Rubio et al., 2019). When cotton is subjected to cold stress, spraying exogenous ABA can reduce the damage of free radicals to chlorophyll and promote the synthesis of photosynthetic pigments to protect the photosynthetic system and improve the cold tolerance of leaves (Li et al., 2019). Under water stress, increased ABA content in leaves promotes K^+^ export and closure of guard cell pores, thereby reducing transpiration and water loss, and improving drought tolerance of leaves (Kano et al., 2011; Wang et al., 2011). In wheat, exogenous ABA was found to have different mechanisms to improve salt and alkali tolerance. Under salt stress, exogenous ABA can reduce the accumulation of Na^+^ and increase K^+^/Na^+^ and Ca^2+^/Na^+^ to alleviate the damage of salt stress, while exogenous ABA alleviates the damage from alkali stress by increasing the synthesis of soluble sugar and citric acid (Li and Mu, 2017). Other studies have shown that the ABA treatment enhances the activity of antioxidant enzymes and drought stress resistance in sugarcane and cotton seedlings (Yao et al., 2005; Li et al., 2010). In addition, ABA enhances the antioxidant capacity of cucumber leaves under Ca(NO_3_)_2_ stress by inducing H_2_O_2_ accumulation (Sheng et al., 2016).

Calcium can reduce the damage of environmental stress to plants (Yan M et al., 2022). Studies have demonstrated that exogenous Ca^2+^ positively regulates the germination rate of *Hulthemia berberifolia* seeds under salt stress (Yan Z et al., 2022). A study of *Gleditsia sinensis* grown in saline-alkali soil showed that exogenous Ca^2+^ can improve the survival rate of *Gleditsia sinensis* by reducing the cytotoxicity caused by salt stress and maintaining the ionic balance *in vivo* (Guo et al., 2021). Under low-temperature stress, exogenous Ca^2+^ can improve the cold tolerance and photosynthetic carbon assimilation in peanut by maintaining effective transport of non-structural carbohydrates and reducing excessive reactive oxygen species accumulation (Liu et al., 2022). Under the stress of low temperatures at night, exogenous Ca^2+^ also protects the peanut photosystems from photoinhibition and supports the integrity of the chloroplast structure, which guarantees the continued export of carbohydrates from the leaves to promote plant growth (Wu et al., 2020).

Tomato is sensitive to drought, especially at the germination and seedling stages. Drought stress induces changes in plant gene expression patterns. Reducing drought damage to seedlings is of great significance for tomato cultivation and production. In this study, we used RNA-sequencing (RNA-seq) to detect transcriptional changes in tomato seedlings in response to drought. The differentially expressed genes (DEGs) were analyzed to examine the response patterns in tomato under different stress environments. Our study reported the response of tomato to exogenous ABA and Ca^2+^ under drought stress and identified a key role for CNGC proteins in regulating the drought resistance pathway in tomato. These results provide basic data for a comprehensive understanding of the drought resistance mechanism in tomato, and new ideas for how to improve the drought resistance in this economically important plant species.

## 2 Materials and methods

### 2.1 Plant materials and treatment

The tomato cultivar “Micro-Tom” was used in this research. The seeds were planted in 7-cm diameter plastic pots filled with nutritious soil. Then, the plastic pots were sealed with a single layer of sterile sealing film and transferred to a 26°C/19 °C (day/night) greenhouse with approximately 70% relative humidity. The sterile sealing film was removed when two true leaves appeared. The 5-week-old tomato seedlings were treated with exogenous 10 mmol/L CaCl_2_ (Zhang et al., 2020) and 0.1 mmol/L ABA (Yan M et al., 2022). In this experiment, four groups were used: WT (water treatment), PE (10% polyethylene glycol [PEG] 6000 treatment), PC (10% PEG 6000 and exogenous Ca^2+^ treatment), and PA (10% PEG 6000 and exogenous ABA treatment). Seedlings were watered with a 1/2 full nutrient solution containing 10% PEG 6000 to simulate drought stress. When tomato seedlings were drought treated for 24 h, exogenous CaCl_2_ or ABA was sprayed at 09:00 and 16:00 every day for five consecutive days. Each treatment was applied until the surfaces of the leaves were coated with small beads of treatment solution.

### 2.2 RNA-seq library construction and sequencing

Total RNA was extracted from four biological replicates per group. Concentration and purity detection of extracted RNA was done using a NanoDrop (NanoDrop 2000; Thermo Scientific, United States). RNA integrity was examined by agarose gel electrophoresis and RNA integrity number (RIN) was determined (Agilent2100 Bioanalyzer System, Agilent Technologies, United States). The mRNA with polyA at the 3’ end was enriched from the total RNA using Oligo (dT) magnetic beads. The mRNA was randomly fragmented in fragmentation buffer and small fragments of about 300 bp were separated using magnetic bead screening. The reverse transcriptase and random primers were added to initially synthesize cDNA using mRNA as a template. Then, a second cDNA strand was synthesized by adding DNA polymerase I and RNase H. End Repair Mix was added to change the sticky ends of the double-stranded cDNA into blunt ends, and then an “A” base was added to the 3ʹ end of the double-stranded cDNA molecule. The product was purified by agarose gel electrophoresis and amplified by PCR. Finally, the Illumina HiSeq 2500 sequencer was used to sequence 16 libraries. The read length was 2 × 150 bp.

### 2.3 Sequencing reads and DEG analysis

In the original sequencing data, the quality of the subsequent assembly was significantly affected by the joint sequence, low-quality read segment, sequence with a high N rate, which indicated an uncertain bias, and excessively short sequences. To ensure the accuracy of subsequent bioinformatics analysis, the original sequencing data were filtered to obtain high-quality clean data. The specific steps were as follows: (1) The joint sequence in the reads and the reads without inserted fragments due to the self-connection of the joining were removed. (2) The bases with a mass value less than 30 at the 3’ end of the sequence were cut out. If there were bases with a mass value of less than 10 in the remaining sequence, the whole sequence was removed. (3) The reads with an N ratio of more than 10% were removed. (4) After pruning, sequences of less than 50 bp were removed.

Sequence alignment between the filtered sequence and the GCF_000188,115.4 *S. lycopersicum* reference genome (https://www.ncbi.nlm.nih.gov/genome/?term=Solanum+lycopersicum) was analyzed using TopHat2 software (https://tophat.cbcb.umd.edu/) (Kim et al., 2013). Gene expression levels were calculated based on the gene length and sequencing depth, and reads were mapped to the genome and normalized to the number of reads per million from each kilobase length of a gene (Li and Dewey, 2011). The DEGseq software was used to analyze the DEGs between groups (Wang et al., 2010). The significance threshold for differential expression was log_2_ |fold change (FC) | ≥ 1 and FDR <0.05.

### 2.4 Gene ontology (GO) and kyoto encyclopedia of genes and genomes (KEGG) functional enrichment analysis

GO analysis organized genes into three categories: cell composition (CC), biological process (BP), and molecular function (MF) to predict their functions. In this study, GO enrichment analysis of the identified genes was studied using the Goatools software (Tang et al., 2015). To explore the cellular metabolic pathways regulated by these genes, we conducted pathway analysis. KEGG pathway enrichment analysis was performed by comparing the genes in the gene sets within the KEGG database. Gene enrichments with *p*-value <0.05 were significant.

### 2.5 Weighted gene co-expression network analysis (WGCNA)

The expression level of 16 transcriptome samples (four groups with four replicates in each group) was analyzed with the WGCNA v 1.70 package (http://horvath.genetics.ucla.edu/html/CoexpressionNetwork/Rpackages/WGCNA/). The network type was unsigned and the lowest correlation between module members and genes was 0.3, and the minimum gene number of the module was 20. The correlation between samples and modules was analyzed with the Pearson correlation coefficient.

### 2.6 Quantitative real-time PCR

Total RNA was isolated from “Micro-Tom” tomato samples using the RNAprep pure Plant Kit (Tiangen Biotech, Beijing, China). Three biological replicates were used for RNA extraction. The mRNA was then transcribed into cDNA using the Fasting RT kit (Tiangen Biotech, Beijing, China). Quantitative real-time PCR was performed using GoTaq qPCR Master Mix (Promega, Beijing, China) in the ABI QuantStudio 6 Flex system with the following conditions: 95°C/10 min (1 cycle); 95°C/15 s, and 60°C/1 min (40 cycles); 95°C/15 s, 60°C/15 s, and 95°C/15 s (1 cycle). Three biological replicates were used in each qRT-PCR experiment. *SlEFα* was used as an internal reference. The relative expression of genes was calculated by using 2^−ΔΔCt^method. The primer pairs are listed in Supplementary Table S1.

## 3 Results

### 3.1 Statistics and quality verification of transcriptome sequencing data from “Micro-Tom” tomato

**FIGURE 1:**
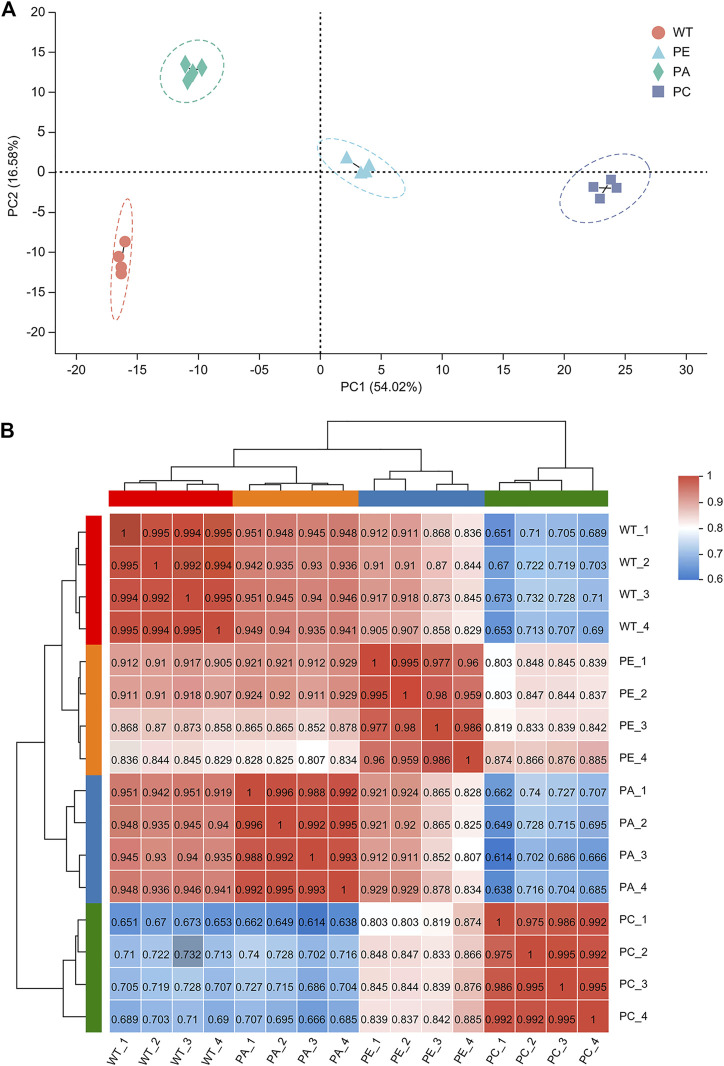
Analysis of sample correlation. **(A)** A principal component analysis (PCA) of the gene expression profiles of each sample. WT (orange circles): four wild-type samples; PE (blue triangles): four drought stress samples; PA (green diamonds): four samples sprayed with exogenous abscisic acid under drought stress; PC (purple squares): four samples sprayed with exogenous calcium under drought stress. **(B)** A heatmap of the Pearson’s correlation among 16 tomato samples. WT_1, WT_2, WT_3 and WT_4 represent WT; PE_1, PE_2, PE_3, and PE_4 represent PE; PA_1, PA_2, PA_3 and PA_4 indicate PA; and PC_1, PC_2, PC_3 and PC_4 indicate PC. Different colored squares indicate the degree of correlation between the two samples. The darker the red color, the higher the correlation between the two samples. The darker the blue color, the lower the correlation between the two samples.

To determine the effects of exogenous ABA and Ca^2+^ on gene expression in tomato under drought stress, we compared the mRNA expression levels of tomato. To avoid sample bias, four biological replicates were collected from each group, with a total of 16 sample data points obtained for RNA-seq library construction and the *S. lycopersicum* SL3.0 assembly used as the reference genome. As shown in Table 1, at least 45.37 million clean reads were obtained, and there were over 7.37 Gb of clean data collected for each sample. The reads with a quality value ≥ Q20 accounted for more than 98% of the total number of reads. The Q30 base distribution percentage ranged from 94.59% to 95.36%. The clean reads were aligned to the tomato reference genome for each sample and the alignment rate was between 95.02% and 96.28%. The clean reads at unique positions in the tomato reference genome accounted for more than 92.98% of the total reads collected. Principal component analysis (PCA) showed that the similarity between samples in each group was high, with obvious separation between groups, which verified the reproducibility of the biological samples (Figure 1A). The correlation between the 16 samples was presented in the form of a heatmap (Figure 1B), which reflected that the higher the correlation coefficient between samples, the more similar the gene expression level. These results further emphasized the high repeatability and reliability of the samples and verified the rationale of the experimental design (Figure 1B).

**TABLE 1:** Statistics of the constructed *Solanum lycopersicum* transcriptome database.

Sample	Raw reads (M)	Clean reads (M)	Clean Bases (Gb)	Clean reads ratio (%)	Q20 (%)	Q30 (%)	Mapped reads (total)	Mapped ratio (total)	M (%)apped reads (uniquely)	Mapped ratio (uniquely)
W (%)T_1	46.29	46.01	6.84	99.41	98.51	95.23	44.01	95.64	43.13	93.74
WT_2	54.45	54.13	8.03	99.41	98.45	95.07	51.80	95.69	50.74	93.74
WT_3	50.68	50.37	7.48	99.39	98.48	95.17	48.29	95.86	47.31	93.92
WT_4	50.34	50.08	7.45	99.49	98.54	95.3	48.06	95.97	47.11	94.06
PE_1	53.09	52.77	7.69	99.39	98.54	95.34	50.62	95.93	49.64	94.08
PE_2	48.35	48.02	7.04	99.31	98.38	94.95	45.95	95.69	45.05	93.81
PE_3	54.07	53.65	7.92	99.22	98.37	94.88	51.38	95.78	50.34	93.84
PE_4	53.04	52.70	7.70	99.37	98.5	95.23	50.63	96.06	49.59	94.09
PA_1	47.55	47.25	7.02	99.38	98.51	95.24	45.25	95.78	44.39	93.94
PA_2	52.72	52.45	7.78	99.47	98.47	95.14	50.09	95.50	49.08	93.59
PA_3	48.52	48.15	7.13	99.23	98.46	95.15	45.96	95.46	44.95	93.36
PA_4	45.64	45.37	6.73	99.41	98.45	95.09	43.41	95.69	42.58	93.86
PC_1	48.21	47.82	6.99	99.20	98.47	95.19	46.05	96.28	45.08	94.26
PC_2	51.97	51.26	7.42	98.62	98.13	94.59	48.70	95.02	47.66	92.98
PC_3	52.12	51.76	7.47	99.31	98.54	95.36	49.81	96.23	48.78	94.24
PC_4	50.48	50.09	7.25	99.23	98.52	95.28	48.05	95.93	46.99	93.81

WT_1, WT_2, WT_3, and WT_4 represent the four wild-type samples; PE_1, PE_2, PE_3, and PE_4 represent the drought stress samples; PA_1, PA_2, PA_3, and PA_4 indicate the samples sprayed with exogenous abscisic acid under drought stress; and PC_1, PC_2, PC_3, and PC_4 indicate the samples sprayed with exogenous calcium under drought stress.

### 3.2 Statistical analysis of DEGs associated with exogenous ABA and calcium treatment during drought stress

**FIGURE 2:**
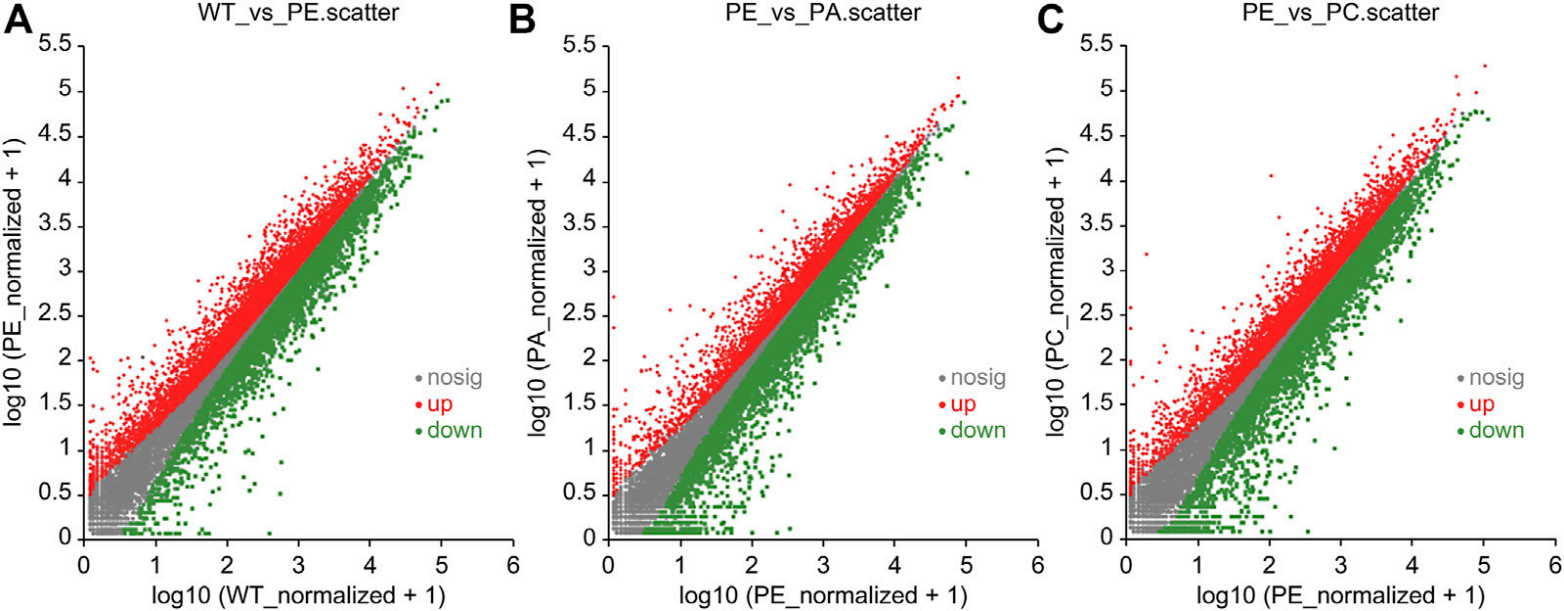
Scatter plots of differentially expressed genes (DEGs). Scatter plots of the **(A)** WT_vs._PE, **(B)** PE_vs._PA, and **(C)** PE_vs._PC comparison groups, respectively. The dots represent specific genes. The abscissa value is the gene expression level in the control group, and the ordinate value is the gene expression level in the treatment group. The red dots represent significantly upregulated genes, the green dots represent significantly downregulated genes, and the gray dots indicate genes that were not significantly different. The closer the point is to 0, the lower the gene expression. The farther the point is from the diagonal, the greater the difference in gene expression between the two compared groups.

**FIGURE 3:**
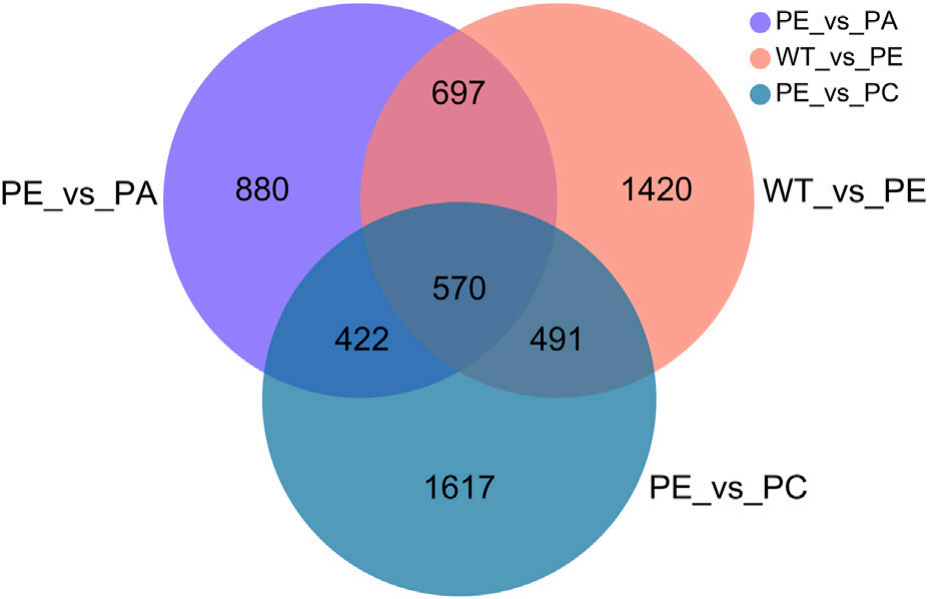
Venn diagram of the DEGs. Results from the WT_vs._PE, PE_vs._PA and PE_vs._PC comparison groups were used for Venn analysis. Different colored circles represent the different comparison groups. WT_vs._PE: pink circle; PE_vs._PA: purple circle; PE_vs._PC: blue circle. The numbers indicate the genes that are common and unique to the different comparison groups. The sum of all the numbers inside the circle represents the total number of DEGs in the comparison group. The intersecting areas of the circles indicate the genes shared between the comparison groups.

The DEGs associated with exogenous ABA and Ca^2+^ treatment were identified in tomatoes grown under drought stress using DESeq2 software (Table 2; Figure 2). The most DEGs were identified in the WT_vs._PE comparison group, containing 12,896, among which 6552 genes were upregulated and 6344 genes downregulated (Table 2; Figure 2A). In the PE_vs._PA comparison group, 11,406 DEGs were identified, with 5589 and 5817 up- and downregulated genes, respectively (Table 2; Figure 2B). A total of 12,502 DEGs were detected in the PE_vs._PC comparison group, among which the expression of 6152 genes was upregulated and that of 6344 genes was downregulated (Table 2; Figure 2C). The results showed that the expression of the DEGs in response to drought stress was greatly affected by exogenous Ca^2+^. A Venn diagram was used to illustrate the number of common and unique genes among each comparison group (Figure 3). As shown in Figure 3, there were 570 DEGs present in all three comparison groups, suggesting that these DEGs might be involved in regulating the resistance of tomato to drought stress.

**TABLE 2:** DEGs in comparison groups.

Comparison group	Total DEG	Up	Down
WT_vs._PE	12,896	6552	6344
PE_vs._PA	11,406	5589	5817
PE_vs._PC	12,502	6152	6350

Total DEG: total number of differentially expressed genes; Up: the number of upregulated DEGs; Down: the number of downregulated DEGs.

### 3.3 Functional analysis of DEGs

**FIGURE 4:**
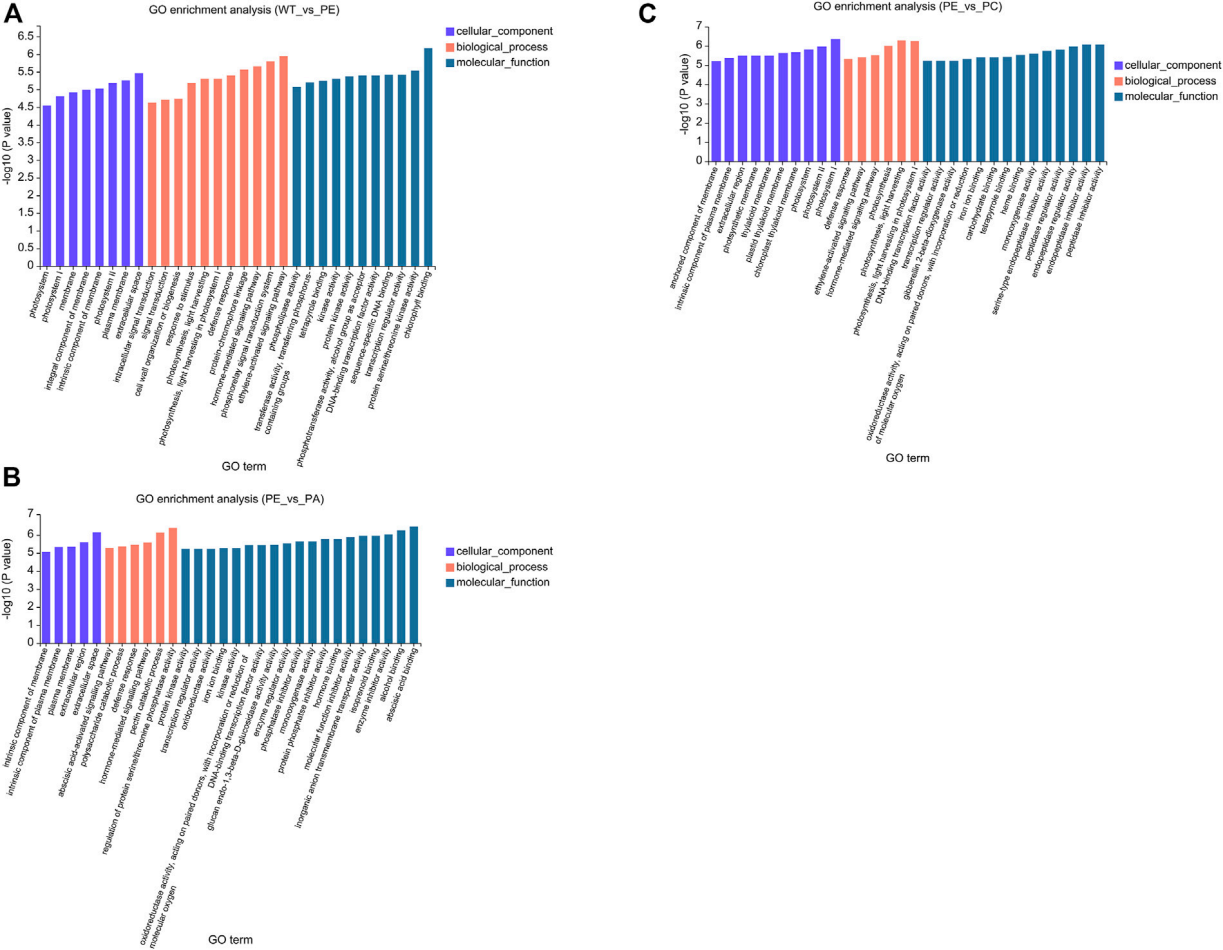
Gene Ontology (GO) functional enrichment of the DEGs. GO enrichment bar graphs of **(A)** WT_vs._PE, **(B)** PE_vs._PA, and **(C)** PE_vs._PC comparison groups. The abscissa represents the GO term, and the ordinate represents the significance of the enrichment, which corresponds to the height of the column. The larger the -log10 (*p*-value) value, the more significantly enriched the GO term.

To better understand the function of the DEGs associated with the WT_vs._PE, PE_vs._PA, and PE_vs._PC comparison groups, GO (Gene Ontology) and KEGG (Kyoto Encyclopedia of Genes and Genomes) pathway enrichment analyses were performed. The GO enrichment analysis of DEGs was used to generate the enrichment results for the top 30 terms (Figure 4 and Supplementary Table S2). There were differences in the number of genes associated with cell components, biological processes, and molecular function among the three comparison groups. In the WT_vs._PE comparison group, the DEGs were enriched in eight cellular components, 11 biological processes, and 11 molecular functions (Figure 4A and Supplementary Table S2A). The DEGs in the PE_vs._PA comparison group were enriched in five cellular components, six biological processes, and 19 molecular functions (Figure 4B and Supplementary Table S2B). The GO enrichment analysis of DEGs in the PE_vs._PC comparison group had 10 genes in cell components, six in biological processes, and 14 in molecular functions (Figure 4C and Supplementary Table S2C). However, those within the significant enrichment in cellular components, biological processes, and molecular functions categories were similar among the three comparison groups. “Cytoplasmic membrane,” “extracellular region,” and “photosystem membrane” were the main terms in cell composition; in biological processes, the DEGs were mainly enriched in “cell communication,” “defense response,” and “photosynthesis”; and in molecular function, the DEGs were enriched in “binding” and “catalytic activity” (Figure 4 and Supplementary Table S2).

**FIGURE 5:**
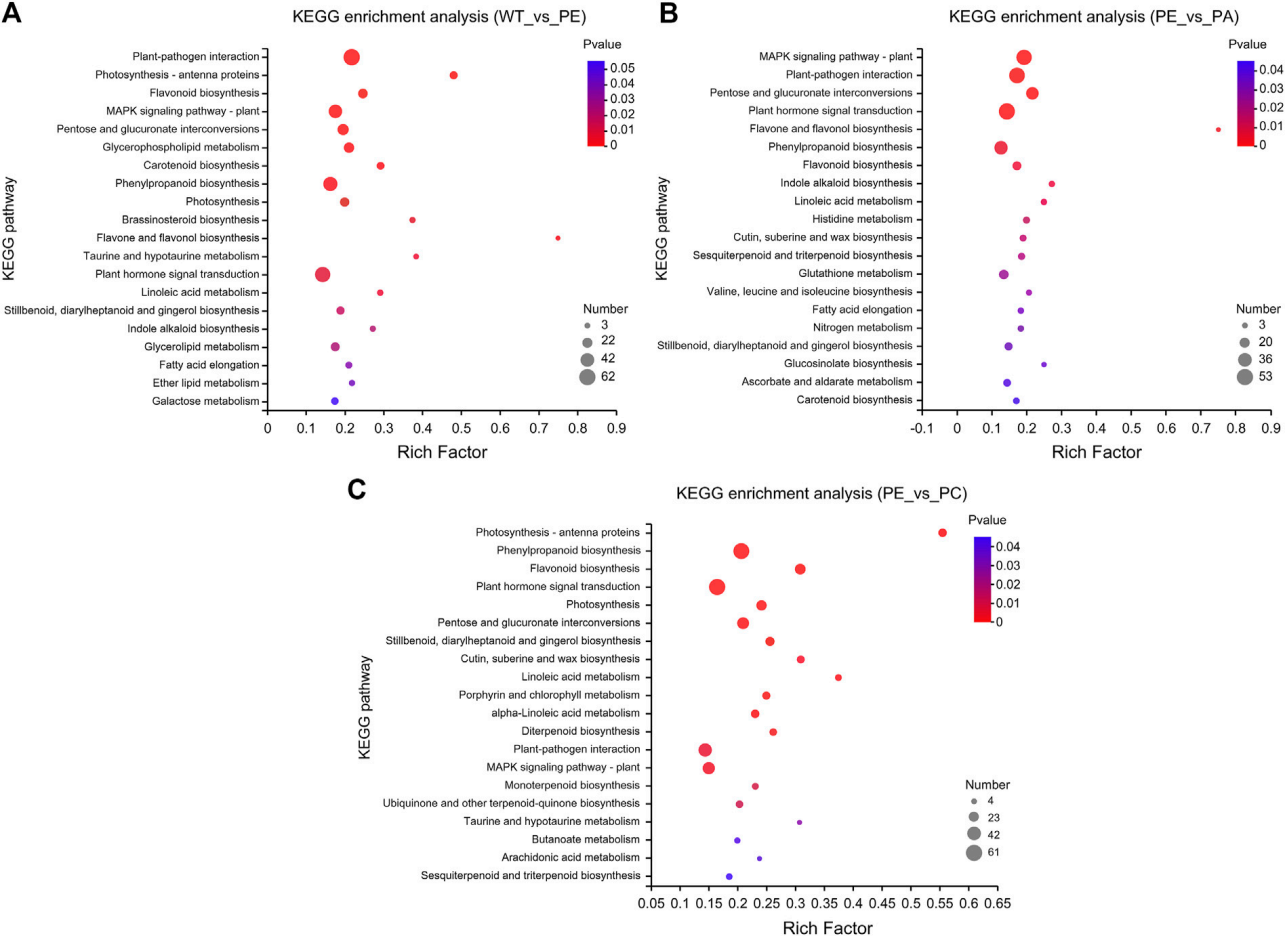
Kyoto encyclopedia of genes and genomes (KEGG) pathway enrichment of DEGs. KEGG pathway enrichment bubble charts of **(A)** WT_vs._PE, **(B)** PE_vs._PA, and **(C)** PE_vs._PC comparison groups, respectively. The abscissa represents the enrichment factor, and the ordinate represents the pathway name. The enrichment factor represents the ratio of the DEG numbers annotated in the pathway term to all gene numbers annotated in that pathway term. The greater the enrichment factor, the greater the degree of pathway enrichment. The bubble sizes represent the number of genes enriched in the KEGG pathway. The bubble color represents the *p*-value.

KEGG pathway enrichment analysis indicated that the DEGs in the WT_vs._PE, PE_vs._PA, and PE_vs._PC comparison groups were significantly enriched in 19, 22, and 23 KEGG pathways, respectively (*p*-value <0.05, Figure 5 and Supplementary Table S3). The KEGG pathways with the greatest accumulation of DEGs were similar in the three comparison groups. Among these KEGG pathways, the greatest enrichment of DEGs was found in “plant-pathogen interaction,” “plant hormone signal transduction,” “phenylpropanoid biosynthesis,” “MAPK signaling pathway-plant,” and “pentose and glucuronate interconversions” pathways (Figure 5 and Supplementary Table S3). Therefore, these DEGs are associated with metabolism, signal transduction, and plant-pathogen interactions and jointly regulate the physiological responses to drought stress and resistance in tomato.

### 3.4 Analysis of genes related to the tomato CNGC

The 19 cyclic nucleotide-gated ion channel genes related to calcium ion transport were identified based on GO (http://www.geneontology.org), KEGG (http://www.genome.jp/kegg/), EggNOG (Clusters of Orthologous Groups of proteins, http://www.ncbi.nlm.nih.gov/COG/), NR (Non-Redundant Protein Sequence Database, ftp://ftp.ncbi.nlm.nih.gov/blast/db/), Swiss-Prot (http://web.expasy.org/docs/swiss-prot_guideline.html), and Pfam (protein families database, http://pfam.xfam.org/) and combined with the related references (Table 3 and Supplementary Table S4). As shown in Table 3 and Table 4, in the WT_vs. _PE comparison group, the expression of 11 SlCNGC genes was upregulated under drought stress, of which *LOC101244112*, *LOC101244669*, *LOC101246478*, *LOC101246872*, and *LOC101248001* were significantly upregulated. After adding exogenous abscisic acid (ABA), the expression of these 11 SlCNGC genes decreased. In addition to *LOC101244669* and *LOC101246478*, the expression levels of the other nine SlCNGC genes were downregulated by exogenous Ca^2+^.

**TABLE 3:** Tomato cyclic nucleotide-gated ion channel gene expression in WT_vs._PE, PE_vs._PA, and PE_vs._PC comparison groups.

Gene name	Gene description	Log_2_ fold-change		
		WT_vs._PE	PE_vs._PA	PE_vs._PC
CNGC5	cyclic nucleotide-gated ion channel 1-like, transcript variant X1	0.65	0.11	0.46
CNGC15	cyclic nucleotide-gated ion channel 15	−0.13	−0.04	−0.55
LOC101244112	protein CNGC15b-like	2.31	−1.14	1.00
LOC101244669	cyclic nucleotide-gated ion channel 1-like	1.66	−1.61	1.70
LOC101246478	cyclic nucleotide-gated ion channel 4-like	1.16	0.13	1.25
LOC101246872	cyclic nucleotide-gated ion channel 1-like	2.02	−0.71	−1.30
LOC101248001	putative cyclic nucleotide-gated ion channel 8, transcript variant X2	2.51	−2.43	−0.82
LOC101248443	cyclic nucleotide-gated ion channel 18	0.40	−0.88	−1.08
LOC101249432	protein CNGC15b, transcript variant X2	0.86	−0.85	−0.35
LOC101249567	probable cyclic nucleotide-gated ion channel 14, transcript variant X4	−0.23	0.83	−1.05
LOC101250084	cyclic nucleotide-gated ion channel 1	0.45	−0.33	−0.26
LOC101250219	cyclic nucleotide-gated ion channel 1, transcript variant X1	0.82	−0.59	0.26
LOC101250508	cyclic nucleotide-gated ion channel 1-like, transcript variant X1	0.85	−0.50	0.12
LOC101251271	probable cyclic nucleotide-gated ion channel 16	−0.32	0.47	−0.09
LOC101263426	cyclic nucleotide-gated ion channel 17	−0.05	0.12	−0.81
LOC101265895	cyclic nucleotide-gated ion channel 4	−0.17	−0.08	1.25
LOC101266887	cyclic nucleotide-gated ion channel 2, transcript variant X1	−2.08	1.82	−0.27
LOC101267093	probable cyclic nucleotide-gated ion channel 5	−0.50	0.20	−0.52
LOC101267716	probable cyclic nucleotide-gated ion channel 5, transcript variant X1	−0.09	−0.17	−0.03

**TABLE 4:** Number of differentially expressed tomato cyclic nucleotide-gated ion channel genes in the treatment comparison groups.

WT_vs._PE		Exogenous substance	Up	Down
Up	11	ABA (PE_vs._PA)	0	11
		Ca^2+^ (PE_vs._PC)	2	9
Down	8	ABA (PE_vs._PA)	7	1
		Ca^2+^ (PE_vs._PC)	4	4

Up: upregulates the number of comparison group; Down: downregulates the number of comparison group; ABA (PE_vs._PA): exogenous ABA, was applied under drought stress; Ca^2+^ (PE_vs._PC): exogenous Ca^2+^ was applied under drought stress.

Eight SlCNGC genes were downregulated in the WT_vs._PE comparison group (Table 3; Table 4), while exogenous ABA treatment enhanced the negative regulation of *LOC101267716* in the drought stress pathway. The other seven genes were upregulated in the PE_vs. _PA comparison group. In the PE_vs._PC comparison group, exogenous Ca^2+^ upregulated the expression of four SlCNGC genes (*LOC101251271*, *LOC101265895*, *LOC101266887*, and *LOC101267716*) and downregulated four SlCNGC genes (*CNGC15*, *LOC101249567*, *LOC101263426* and *LOC101267093*). These results indicated that exogenous ABA inhibited the regulation of most SlCNGC genes in the drought stress pathway, while exogenous Ca^2+^ only enhanced or inhibited the regulation of some SlCNGC genes.

### 3.5 WGCNA of DEGs

**FIGURE 6:**
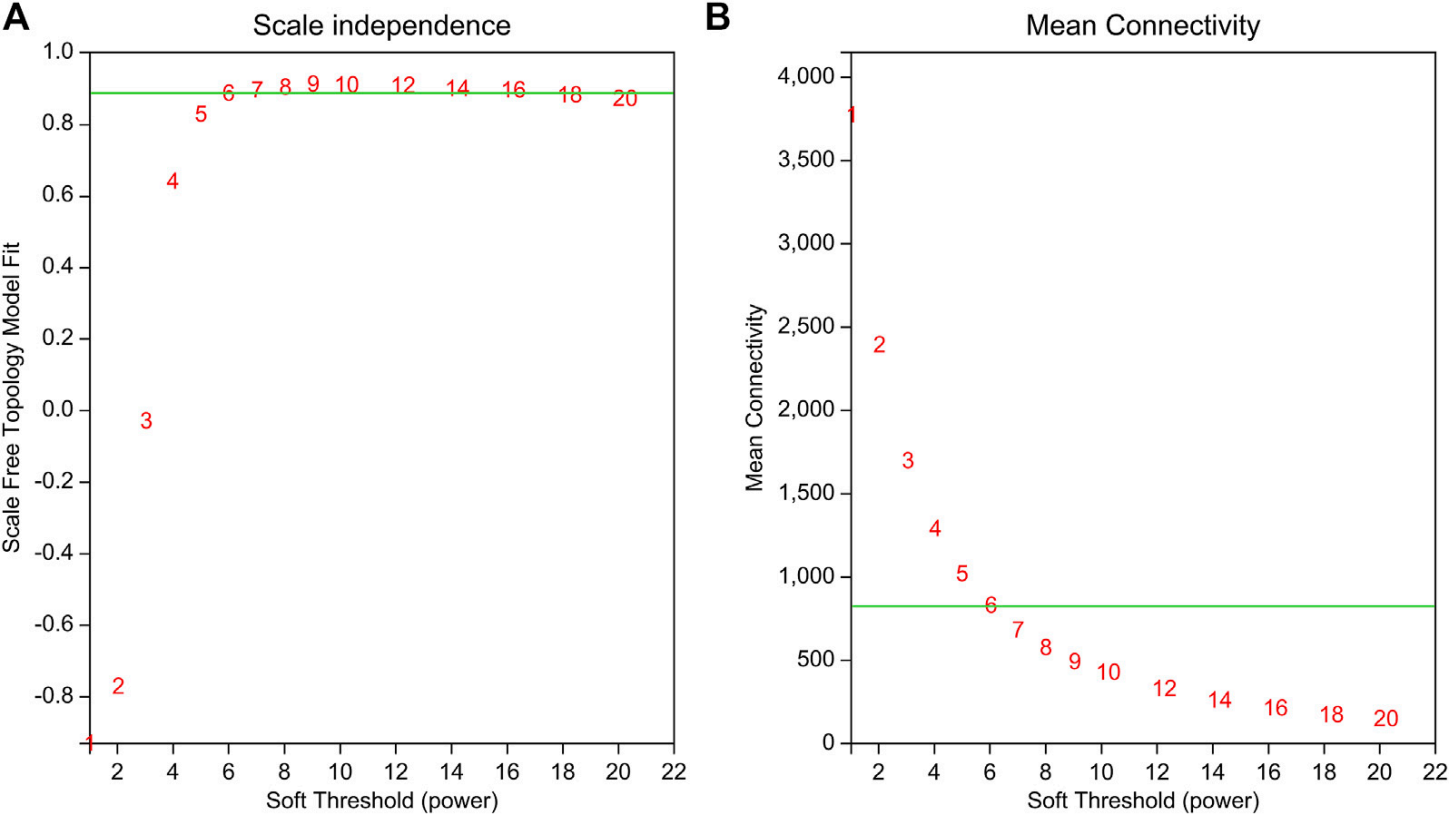
Determination of the power beta (β) value based on the adjacency matrix using Weighted gene co-expression network analysis (WGCNA). The weighted parameter power β value was determined using the scale-free topology criterion. Based on the two graphs of **(A)** topology fitting results and **(B)** mean connectivity, we choose β = 6 to ensure that the average connectivity of the network was smooth.

**FIGURE 7:**
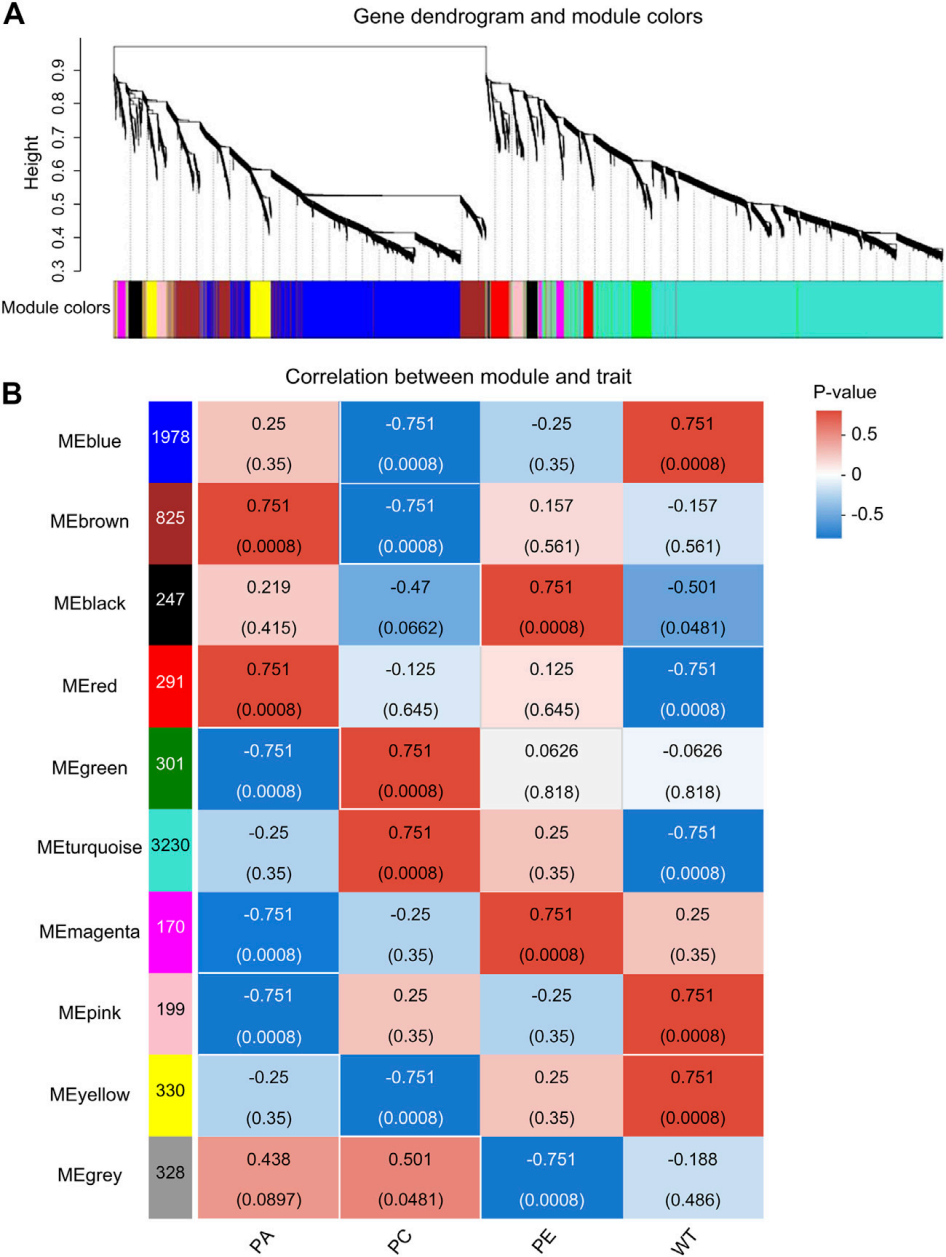
Analysis of expression patterns in tomato samples by WGCNA. **(A)** A module classification tree. Each branch of the cluster tree represents a module, with each leaf representing a gene. Each color represents a module. If the color is gray, the genes did not divide into specific modules. **(B)** A module-sample association heatmap. The abscissa represents the different samples, and the ordinate represents the different modules. The column on the left represents the number of genes in the module, while each group of data on the right represents the correlation coefficient (r) and *p*-value (in parentheses) between the gene in the module and the sample. Red and blue represent positive and negative correlations between modules and samples, respectively.

WGCNA is a common tool for analyzing the relationships between large numbers of genes. The gene expression patterns were similar in the same gene co-expression network, but the gene expression patterns were different in different gene co-expression networks. A scale-free grid was constructed, which showed that the average connectivity of the network was smooth when the weighted parameter power β = 6 (Figure 6). The WGCNA algorithm was used to analyze gene clustering and module partitioning of the 16 samples collected in the drought stress experiment. As observed in the dendrogram (Figure 7A), ten gene co-expression modules were obtained, with the number of genes in the modules ranging from 170 (magenta) to 3230 (turquoise) (Figure 7B; Table 5). The greater the correlation (r) between the module and the sample, the higher or lower the gene expression in the module. We identified three high-expression and two low-expression gene modules in the WT group, two high-expression and one low-expression gene modules in the PE group, and two high-expression gene modules and three low-expression gene modules in the PC and PA groups, respectively (*r* > 0.7; Figure 7B).

**TABLE 5:** Number of genes in each module identified using WGCNA.

Module	Number of genes
Blue	1,978
Brown	825
Turquoise	3,230
Gray	328
Yellow	330
Pink	199
Black	247
Green	301
Magenta	170
Red	291

Module colors correspond to those used in Figure 7B.

By analyzing the correlation coefficients of each module, we found that the genes in the MEbrown and MEred modules showed higher specificity in PA samples, while the genes of MEbrown and MEturquoise modules showed higher specificity in PC samples when compared with other samples (Figure 7B). The *LOC101249567* candidate gene was located in the MEbrown module, while *LOC101244669*, *LOC101246478*, *LOC101265895*, and *LOC101249432* candidate genes were located in MEturquoise module.

### 3.6 Functional enrichment analyses of genes in the MEbrown and MEturquoise modules

**FIGURE 8:**
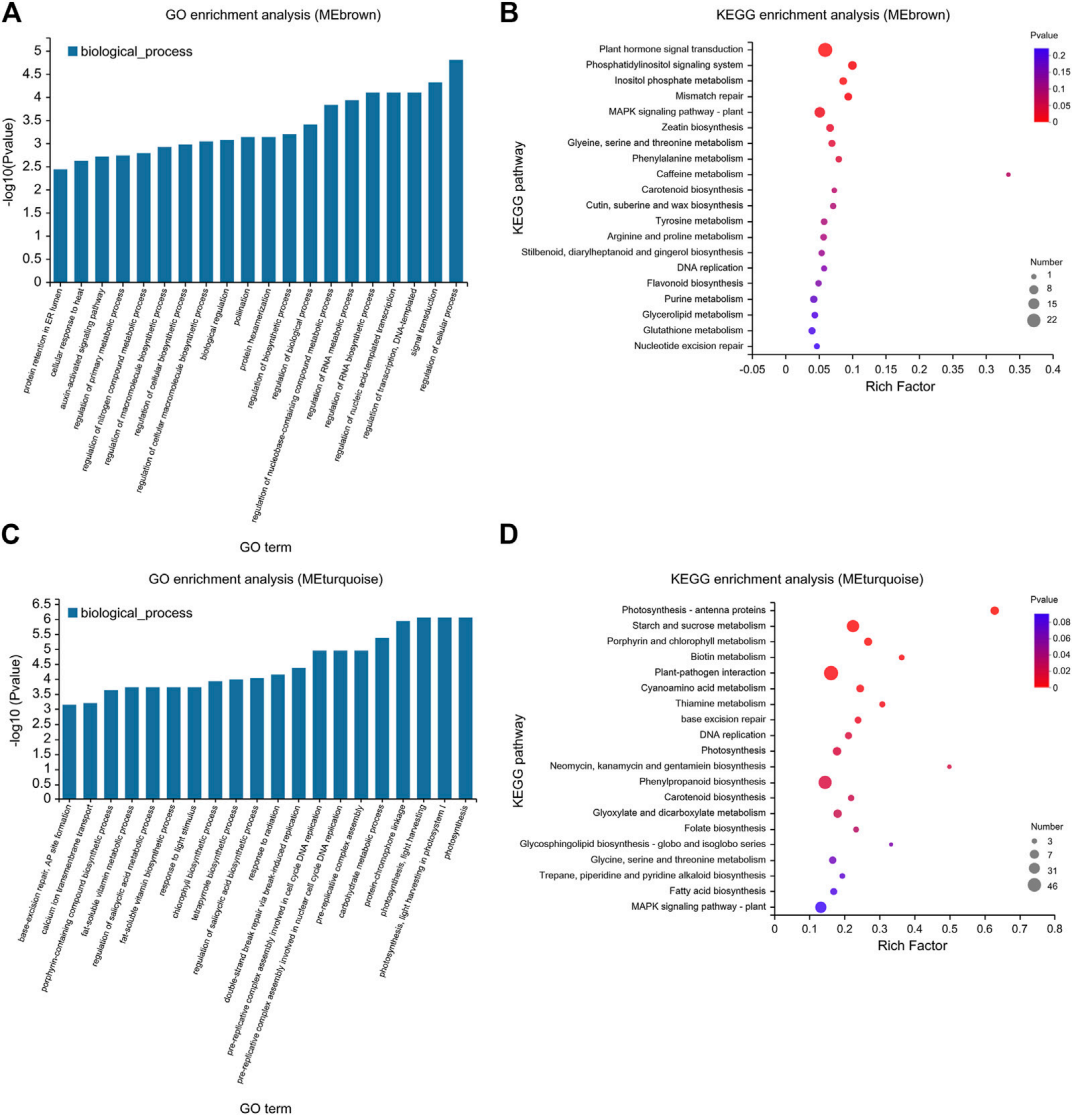
GO and KEGG enrichment map of MEbrown and MEturquoise modules using the WGCNA. **(A)** GO and **(B)** KEGG pathways enriched in the MEbrown module. **(C)** GO and **(D)** KEGG pathways enriched in the MEturquoise module. **(A,C)** The abscissa represents GO terms, and the ordinate represents the enrichment significance level, which corresponds to the height of the column. The larger the -log10 (*p*-value) value, the more significantly enriched the GO term. **(B,D)** The enrichment factor is defined as the ratio of the DEGs annotated in a pathway term to all the genes annotated in that pathway term. The greater the enrichment factor, the greater the degree of pathway enrichment. The bubble sizes represent the number of genes enriched in a KEGG pathway. The bubble color represents the *p*-value.

To explore the response of tomato to exogenous ABA and Ca^2+^ under drought stress, GO and KEGG pathway enrichment analyses of MEbrown and MEturquoise modules were performed, and the top 20 genes were identified (Figure 8). Based on the GO analysis, in the MEbrown module, highly enriched biological processes included auxin signal transduction, macromolecular biosynthesis, regulation of the nucleobase-containing compound metabolic process, RNA metabolism, and regulation of primary metabolic processes (Figure 8A). KEGG pathway analysis of these genes showed they were mainly enriched in primary metabolic process, secondary metabolite synthesis, and plant hormone signal transduction (Figure 8B). GO enrichment analysis of the MEturquoise module showed that MEturquoise module genes were involved in various biological processes such as fat-soluble vitamin biosynthesis and metabolism, chlorophyll biosynthesis, salicylic acid biosynthesis and metabolism, porphyrin-containing compound biosynthesis, DNA replication, carbohydrate metabolism, and photosynthesis. Furthermore, these genes were enriched in KEGG pathways included amino acid metabolism, water-soluble vitamin biosynthesis and metabolism, DNA replication, starch and sucrose metabolism, porphyrin and chlorophyll metabolism, and photosynthesis (Figures 8C, D).

### 3.7 Validation of RNA-seq data by RT-qPCR

**FIGURE 9:**
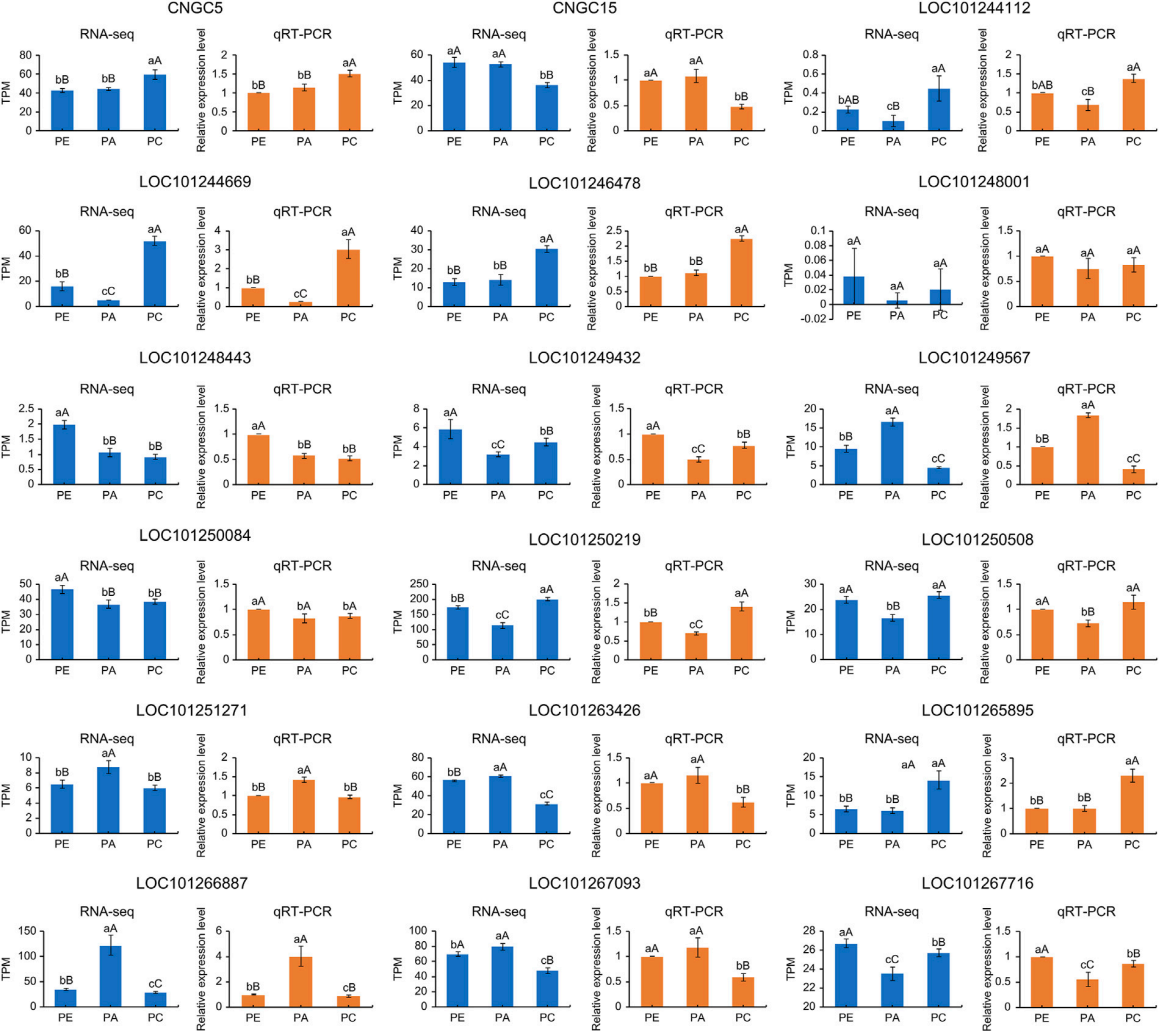
TPM values and relative expression levels of ten cyclic nucleotide-gated ion channel genes expressed in PE, PA, and PC. The orange bar graphs show gene expression measured by qRT-PCR and the blue bar graphs show gene expression measured by RNA-seq in TPM unit. WT: wild-type samples; PE: the drought stress samples; PA: the samples sprayed with exogenous abscisic acid under drought stress; PC: the samples sprayed with exogenous calcium under drought stress. Data are means (±SD) of three biological replicates. Different lowercase and uppercase letters indicate significant differences at *p* < 0.05 and *p* < 0.01, respectively.

We randomly selected ten cyclic nucleotide-gated ion channel genes to verify the accuracy of RNA-seq results by RT-qPCR. The results showed that the relative expression levels of the samples were consistent with the RNA-seq results (Figure 9). Fold changes that differed between the RT-qPCR and RNA-seq data could reflect differences in sensitivity and specificity between RT-qPCR and high-throughput sequencing techniques. Nevertheless, these results demonstrated the reproducibility and reliability of RNA-seq data.

## 4 Discussion

In nature, plant growth is affected by external environmental and endogenous factors. When exposed to harsh environments, plants change their morphology and physiological processes to resist various risks to their survival. As a key endogenous factor mediating plant stress response, plant hormones integrate coping mechanisms with environmental stimuli, which can alleviate the damage caused by stresses such as drought, directly enhancing environmental adaptability and stress resistance in crop plants (Bari and Jones, 2009; Nakashima and Yamaguchi-Shinozaki, 2013). Therefore, in addition to breeding superior varieties with strong stress resistance, researchers are trying to treat seedlings with appropriate exogenous substances to improve their adaptability to the environment. The regulation of cytosolic Ca^2+^ concentration and its response to internal and external stimuli occur throughout the plant life cycle. This regulation is necessary for plant growth and development and for their resistance to biotic and abiotic stresses (Hirschi, 2004; Taneja et al., 2016). CNGC are good candidates for plasma membrane Ca^2+^-permeable influx channels in plants (McAinsh and Pittman, 2009). To investigate how SlCNGC plays a regulatory role in tomato under drought stress and their responses to exogenous calcium and ABA, we sequenced the transcriptome of four treatment groups (WT, PE, PA, and PC) and verified the transcriptome data by qRT-PCR.

The results showed that the data obtained by RNA-seq were reliable and identified 12,896, 11,406, and 12,502 DEGs in the WT_vs._PE, PE_vs._PA, and PE_vs._PC groups, respectively (Table 2). Based on the DEG analysis, we found that some genes have different responses to different stresses, and that gene expression can change depending on the growth environment (Table 2, Figures 2, 3). We identified 19 SlCNGC genes related to calcium ion transport (Table 3), which was inconsistent with the results of a previous study in which 18 SlCNGC genes were identified in tomato (Saand et al., 2015). Our results showed that 11 SlCNGC genes were upregulated and 8 were downregulated under drought stress when compared with those in wild-type tomato. In particular, exogenous ABA inhibited the regulation of most SlCNGC genes associated with the drought stress pathway, while exogenous calcium only enhanced or inhibited the regulation of some of these SlCNGC genes (Table 4). Application of ABA and Ca^2+^ both can increase tomato drought endurance. However, the CNGCs genes in response to exogenous ABA and Ca^2+^ were exhibited with diverse difference, that is, 8 SlCNGC genes (*CNGC15*, *LOC101249567*, *LOC101251271*, *LOC101263426*, *LOC10126589*5, *LOC101266887* and *LOC101267093*) that were upregulated after exogenous ABA application, and 6 SlCNGC genes (*LOC101244669*, *LOC101246478*, *LOC101251271*, *LOC101265895*, *LOC101266887* and *LOC101267716*) that were upregulated after exogenous Ca^2+^ application, it suggested that ABA promoted gene expression and may increase drought resistance in tomato. In the GO enrichment analysis, “cytoplasmic membrane,” “extracellular region,” and “photosystem membrane” were the main terms within the cell composition category. In biological processes, DEGs were mainly enriched in “cell communication,” “defense response,” and “photosynthesis.” In the molecular function category, DEGs were enriched in both “binding” and “catalytic activity” (Figure 4). Among these KEGG pathways, although the selected comparison groups were different, the greatest enrichment of DEGs was found in “plant-pathogen interaction,” “plant hormone signal transduction,” “phenylpropanoid biosynthesis,” “MAPK signaling pathway—plant,” and “pentose and glucuronate interconversions” (Figure 5). These pathways are closely related to plant stress regulation and consistent with the genes being associated with drought stress response and tolerance.

CNGC is considered to be the upstream medium of calcium signal transduction (Gao et al., 2014). CNGC proteins are activated by the direct binding of nucleotides, such as cAMP and cGMP, to the CNBD domain and inhibited by the binding of CaM to the CaMBD domain (Zhou et al., 2014). These proteins are located in the plasma membrane as monomers, where they transport extracellular calcium ions into the cell and then combine with CaM to transmit downstream signals (Zhou et al., 2014; Wang et al., 2019; Wang X et al., 2021). As an important calcium channel in the plasma membrane, CNGC plays an indispensable role in plant growth and response to abiotic stress. At present, the CNGC gene has been well-studied in *A. thaliana*. Studies have shown that AtCNGC2 has various regulatory functions in *A. thaliana*, including pollen tube growth (Fortuna et al., 2015; Wu et al., 2021), flowering transition (Fortuna et al., 2015), leaf senescence (Wang et al., 2017), and auxin synthesis (Chakraborty et al., 2021). Different CNGCs also play similar roles in regulating signal transduction. AtCNGC1, AtCNGC10, AtCNGC14, ATCNGC15, and ATCNGC19 regulate the root growth of *A. thaliana* in different ways. The interaction between AtCNGC14 and AtCaM7 regulates the auxins- and gravity-induced Ca^2+^ signaling pathway to promote rapid root growth of *A. thaliana* (Shih et al., 2015; Brost et al., 2019; Zeb et al., 2020). AtCNGC1 affected the taproot growth of *A. thaliana* seedlings by absorbing Ca^2+^ into the root system (Ma et al., 2006). By interacting with CaM and cGMP, AtCNGC10 regulates cell K+ balance, thereby affecting root geotropism, cell division, and growth of *A. thaliana* (Li et al., 2005; Borsics et al., 2007; Christopher et al., 2007). ATCNGC19 regulates the colonization of *Piriformospora indica* in the roots to promote root growth (Jogawat et al., 2020). The dynamic interaction between ATCNGC15 and nitrate receptor (NRT1.1) regulates ATCNGC15 activity and Ca2+ influx to influence the root growth of *A. thaliana* (Wang D et al., 2021). AtCNGC2 and AtCNGC4 have also been shown to regulate flowering transition in plants on the same signaling pathway (Chin et al., 2013). However, it has also been shown that AtCNGC2 and AtCNGC4 have opposite effects in regulating *A. thaliana* response to heat stress. Chaiwongsar et al. (2009) found that AtCNGC4 may be a part of the hypersensitive signaling pathway in response to heat stress. AtCNGC2 deficiency can enhance the tolerance of *A. thaliana* seedlings to heat stress (Fortuna et al., 2015). Similarly, AtCNGC10 negatively regulates the salt tolerance of plants, and AtCNGC10 overexpression lines are more sensitive to a high-salt environment (Jin et al., 2015). On the contrary, AtCNGC19 expression is upregulated when the local salt concentration increases, which is involved in regulating the toxicity effects caused by salt stress (Kugler et al., 2009).

To our knowledge, we report for the first time the response pattern of SlCNGC genes to exogenous ABA and Ca^2+^ under drought stress. Previous studies provided transcriptome data for tomato response to exogenous ABA (Wang et al., 2013). This study reports the effects of exogenous ABA on the expression of ABA signal transduction-related genes, ABA-induced transcription factors, heat shock protein-related genes, active oxygen scavenging system-related genes, and pathogen resistance-related genes in tomato. However, in a recent report, Zhang et al. provided cDNA-AFLP analysis data of tomato calcium chloride-induced transcripts derived under drought stress, which reported sequence analysis and functional annotation of 34 transcript-derived fragments successfully sequenced under drought stress (Zhang et al., 2020). Saand et al. conducted bioinformatics analysis of SlCNGC genes and studied the response of SlCNGC to *Sclerotinia sclerotiorum* (Ss), *S. sclerotiorum* (Ss)-secreted phytotoxin (OA), *Pseudomonas syringae* pv.tomato (Pst DC3000), *Xanthomonas oryzae* pv.oryzae (Xoo), ethylene (ETH), acibenzolar-S-methyl (BTH), and jasmonic acid (JA) (Saand et al., 2015). However, it does not include the response of SlCNGC to drought stress. The above studies only studied one aspect of tomato and did not specifically study the response of tomato SlCNGC genes to exogenous abscisic acid and calcium under drought stress. Our study fills this gap, which provided meaningful data for breeding resistant varieties and selecting appropriate exogenous substances to enhance the resistance of resistant varieties to drought stress. We will further explore the expression pattern of SlCNGC genes in tomato seedlings treated with combination of ABA and calcium under drought stress, and characterize which one is first or upstream among ABA or calcium influx in signaling.

## Data Availability

The original contributions presented in the study are publicly available. This data can be found here: https://www.ncbi.nlm.nih.gov/ (Accession number PRJNA925706).
